# Women’s views on contact with a health visitor during pregnancy: an interview study

**DOI:** 10.1017/S146342361900046X

**Published:** 2019-07-01

**Authors:** Ellinor K. Olander, Maria Raisa Jessica (Ryc) Aquino, Celine Chhoa, Erica Harris, Suzanne Lee, Ros Bryar

**Affiliations:** 1 Centre for Maternal and Child Health Research, School of Health Sciences, City, University of London, London, UK; 2 Primary Care Unit, Department of Public Health and Primary Care, University of Cambridge, Cambridge, UK; 3 Department of Psychology and Human Development, UCL Institute of Education, University College London, London, UK

**Keywords:** antenatal care, health visiting, interviews, home visit

## Abstract

**Aim:**

To explore recent mothers’ views of the health visiting antenatal contact in England.

**Background:**

English health visitors are mandated to be in contact with all women in the third trimester of pregnancy. The aim of this antenatal contact is to assess the needs of the family before the birth and support preparation for parenthood. Recent data show that this contact is provided fragmentarily and not always face-to-face. More information on how women view this contact could inform service provision.

**Methods:**

Twenty-nine mothers with a baby less than 1 year old were recruited via social media and word of mouth. Having had antenatal contact with a health visitor was not a requirement to participate in the study. Women took part in face-to-face or phone interviews and all recordings were transcribed verbatim. Data were analysed using systematic thematic analysis.

**Findings:**

Eleven women had contact with a health visitor during pregnancy: nine through a home visit, one via a letter and one via a phone call. The remaining 18 women were asked about what they would have wanted from an antenatal contact. Three themes were identified: relationship building, information provision, and mode and time of contact. Some participants who had experienced a home visit reported building rapport with their health visitor before the postnatal period, but not everyone had this experience. Women reported requesting and receiving information about the health visiting service and the role of the health visitor. Finally, women suggested different modes of contact, suggesting a letter or that the information about health visiting could be provided by a midwife. A few women preferred a home visit. These study findings show women were unclear regarding the aim of the health visitor antenatal contact. As such, the contact is unlikely to reach its full potential in supporting parents-to-be.

## Introduction

The antenatal contact by health visitors has been recommended in England since 2009. This was when the Healthy Child Programme supported the contact between 28- and 36-week gestation by health visitors to women and their partners (Department of Health, 2009). With the transfer of commissioning of health visiting to local government in England in 2015, five contacts by health visitors with families, including the antenatal contact, were mandated to ensure continuity of services (Public Health England, 2016). Health visitors are midwives or nurses who have completed additional educational programmes to equip them to provide expert guidance, assessment and interventions to pregnant women and families with children 0–5 years of age (NHS England, 2014). The aim of this antenatal contact is to provide ‘Support with a physical and mental health assessment, preparation for parenthood, guidance on bonding and early child development’ (UNICEF UK, 2017) and is in addition to the routine care women receive from midwifery services.

Health visiting has been said to be the only universal healthcare service that can provide health promotion, early intervention and primary prevention throughout the perinatal period (Christie, 2016). Australian research confirms the importance of nurse home visiting in the antenatal period as it has been found to be associated with positive birth outcomes and helping women to cope and care for their baby postnatally (Kemp *et al*., 2013). Approximately 283 000 antenatal contacts were conducted nationally in 2016/17 (Public Health England, 2017); however, little is known about these contacts. Research to date from England comes from a service evaluation (Monaghan and Owen, 2012), a small survey (Curtis and Davidson, 2013) and an interview study (Donetto *et al*., 2013) all conducted before the antenatal contact was mandated. Taken together, the findings from these studies are mixed. For example, women report wanting to meet a health visitor antenatally (Donetto *et al*., 2013; Curtis and Davidson, 2013) in line with research from Sweden (Barimani and Hylander, 2012). However, when services have been offered no women have attended (Curtis and Davidson, 2013). Other women have questioned the purpose of the contact, wondering why health visitors need to meet with the family before the baby is born (Donetto *et al*., 2013). That said, women who have received an antenatal home visit and continued seeing their health visitor postnatally found this continuity supportive (Donetto *et al*., 2013).

Previous research has also found that the availability of antenatal home visits predicted more postnatal home visits and more frequent community-based activities (Cowley *et al*., 2007). That said, the availability of antenatal contacts is likely to vary. Recent data suggest that approximately 70% of families do not receive a health visitor visit during pregnancy (Astrup, 2017). This uneven provision of services and mixed research evidence underpins our study. The significance of this research was also highlighted by service users through our research centre Patient and Public Involvement (PPI) group. Service users should be consulted when identifying research priorities (Pandey *et al*., 2015) and in our case none of our PPI representatives had had an antenatal contact with a health visitor and described this as a notable omission in their care when the idea for the study was discussed with them. One PPI group member (EH) was part of the research team for the current study to ensure its relevance to current service users. She provided input on initial study protocol, recruitment, discussion of findings and implications for practice.

Based on the above, the intention of this study is therefore to update the literature and explore recent mothers’ experiences and views of the health visiting antenatal contact by interviewing women who had had this contact and those who did not.

## Methodology

### Study background

The current findings come from a large interview study which aimed to explore women’s views on collaboration between midwives and health visitors’ during and after pregnancy. This in turn is part of a larger programme of work aiming to improve healthcare for women and their children by improving the collaboration between healthcare professionals caring for them during and after pregnancy (see https://blogs.city.ac.uk/cope/).

### Participants and recruitment

To be eligible for the study, women had to have had a baby within 12 months prior to the interview. Participants also needed to be able to read and speak English, be over 18 years old, and have had antenatal and postnatal care in England. Having had antenatal contact with a health visitor was not a requirement to participate in the study; however, when we realised very few participants had a health visitor antenatal contact we specifically sought participants who had this experience.

Study participants were recruited through two methods: word-of-mouth and social media. Word-of-mouth recruitment was done by the research team and others affiliated to our research centre and the PPI representative. Social media was used both by the researchers by advertising the study on Twitter and posting in Facebook groups relevant to the population as well as by a large UK charity, the National Childbirth Trust, who advertised the study once through their Facebook page.

Any women interested in participating in the study were asked to email the researchers. The women were then sent a participant information sheet and consent form and an interview was organised. The participants were offered a telephone or a face-to-face interview in London or the West Midlands [where the research assistants conducting the interviews (CC and RA) were based]. Both interviewers were women, educated to MSc level in psychology and had previous experience conducting interviews. Four participants chose face-to-face interviews in their home and the remainder were interviewed by telephone. All women consented to take part in the study before the interview started, verbally if taking part in a phone interview or written consent if a face-to-face interview. None of the interviewers knew the participants before the interview and no one else was present during the interviews.

The part of the interview schedule relevant to this study was informed by previous research (Jenkins *et al*., 2015; Aquino *et al*., 2016) and current policy (Department of Health, 2009; Public Health England, 2013). The present findings focus on the participants answers to the question ‘Did you meet with a health visitor or were in contact with one, when you were pregnant? (If yes, what were your experiences? If no, do you think this would have been helpful? (Why, why not?))’. Based on a suggestion from our PPI representative, a small incentive (£5 Amazon voucher) was offered to participants to thank them for their study participation. This study was approved by the authors’ school ethics committee (Reference PR/MCH/Staff/16-17/02).

### Data analysis

Transcripts were analysed using thematic analysis according to the method proposed by Braun and Clarke (2006). This is a systematic, data-driven method of identifying and organising themes and patterns within data (Boyatzis, 1989). This method facilitates in-depth exploration and understanding of data and facilitates vivid descriptions of participants’ perspectives and experiences (Burns, 1989). Recorded interviews were transcribed by a professional transcription agency and all identifying data were removed. Transcripts were read several times to ensure familiarity with the data. Initial codes arising from the data were identified and these were refined and organised into potential themes. In order to ensure a transparent and auditable analysis process, there was discussion between the research team to ensure there was consensus regarding the themes and their supporting data.

## Results

Forty women contacted the research team, of whom 29 were interviewed in the summer and autumn of 2016. Eleven women chose to not contact the researchers after having received the participant information sheet and consent form. The reasons for this are not known. Data on the participants and the interview length are presented in Table 1. In total, 11 women reported having had contact with a health visitor antenatally. We defined contact as to involve a letter (*n* = 1), phone call (*n* = 1) or face-to-face meeting (*n* = 9). All face-to-face meetings were home visits in this study. All women who reported not having an antenatal contact reported living in greater London (with missing data from three participants) and women who had a contact reported living in greater London (*n* = 4), South West of England (*n* = 2), Midlands (*n* = 2) and North of England (*n* = 1), with missing data from two participants.

**Table 1.** Participant characteristics and interview length

Participant characteristics	Antenatal contact (*n* = 11)	No antenatal contact (*n* = 18)
Mean age of mother (years)	32.54	33.66
First time mother (%)	63.63	66
Age in months of youngest child at time of interview (age range in months)	4 months (1–10)	5.88 months (1–11)
Interview length in minutes (range in minutes)	28 (19–49)	27 (range 16–47)

### Themes

Three clear themes were identified: relationship building, information provision and mode and time of contact. These themes are described below. Quotes related to each theme are presented in italics and participants have been given a fictitious name to ensure their confidentiality. AC after the name means the participant had antenatal contact with a health visitor, no AC means no antenatal contact.

### Relationship building

Several of the participants who had a home visit from their health visitor antenatally spoke positively of this experience. Meeting a health visitor during pregnancy helped with relationship building, knowing who to contact, discussing concerns and building confidence in parenting skills.

It was just a general meet and greet, so because she was just introducing herself and saying that she’d come back after the babies had been born and, trying to think, she gave me her contact details in case I needed to contact her before. I can’t really remember what else she said… Just knowing who she was so that I’d recognise her when she came back, yeah, so it was, I guess it’s part of relationship building isn’t it?

Anna – AC

… it was a nice to see, it was nice to see the same woman, a familiar face, it was nice to have it in my home. I think women, well some women don’t, I quite like having the appointments, it’s quite nice to talk about things and you feel more confident in having that, you feel confident seeing they’re happy and you feel more excited and it’s nice of them to come to your home.

Clara – AC

One participant reported having the same health visitor as for her previous child, ensuring continuity of carer.

Well she actually came to see me before I had the baby … and I actually happened to know her, because she was also my son’s, my second son’s health visitor as well. So there’s continuity, and she came, I think she’s come to the house once after [daughter] been born as well.

Grace – AC

Two participants, however, reported less positive experiences. One participant said she felt the health visitor was checking up on her. Another participant questioned the health visitor’s knowledge and after the visit considered opting out of health visitor appointments.

…she came to the house before the baby was born, that was like the RSPCA visit where they come to check. But she said to me that that’s not what it was about but in your mind you’re like, it is.

Fiona – AC

The health visitor came, I don’t know, maybe when I was about 30 weeks and this was a health visitor that didn’t appear to have a clue about anything. She was a trainee in her defence but she failed to turn up at the correct time and then she basically gave me a pile of leaflets and said, you should know everything because you’ve already got a child. And said things like, oh you breast fed your daughter so therefore you won’t have any problems. Which everybody knows that that’s not necessarily the case… Yeah she was not good and after that I actually spoke to the midwife and she said to me at that point in time, she said, are you aware that you don’t actually have to have health visitor appointments if you’d rather not, you can opt out of them. And I said to her, well I’ll see what the next one is like, but if it’s like that then I think we’ll just say we’re fine to go it alone. But as it was the next lady we saw was perfectly fine.

Donna – AC

### Information provision

Several of the women who had antenatal contact with a health visitor reported that the aim of this seemed to be about providing general information about health visiting. This was not seen as necessary or useful by all women due to receiving this information again postnatally or due to already having had a baby.

While I was pregnant I think I had a letter from the health visiting team, just saying that they were the health visiting team. I think that was the only contact I had with the health visitors before the birth.

Helen – AC

… it was just an introduction into health visitors and what they do. I didn’t really feel like it was necessary because you end up going through, in hindsight you end up going through all of that when you get your first postnatal visit anyway. I didn’t really feel like it was overly helpful to me. I just felt like it was a, it felt very much like a check box that needed to be ticked.

Emma – AC

It’s not like, I can remember [my partner] and I saying, … it’s not like it felt, not necessarily like I felt it was needed so they left and we were like, oh that was nice, it wasn’t like, for us it was hugely beneficial because the second time, might have found it more beneficial the first time with [first child], because you feel a little bit more of an expert at it, so there probably weren’t quite so many questions from my end, have you got everything ready, yes we’ve got everything ready, because you’ve done this before. It had been quite different with [first child].

Clara – AC

The women who had not had antenatal contact with a health visitor consistently reported wanting more information on the role of the health visitor, how it differed from the midwife and what to expect from the health visiting service.

I still am not totally clear on the distinction between a midwife and a health visitor. I understand that a midwife sees you antenatally and then for a couple of weeks postnatally and then hands over to the health visitor but, I think that those early days are a bit blurred really in terms of the distinction between them.

Isabel – no AC

No, I hadn’t met health visitor and to be honest I have to say and I have to check on the internet what the health visitors means because I didn’t know what is the reason they’re coming and what to expect.

Magda – no AC

### Mode and timing of contact

There was little consensus between the participants of what was the best type of health visitor contact. This related back to what the women thought the aim of the contact was, when women believed it was primarily about information about the health visiting service, they suggested a letter from the health visiting team or information from the midwife as good alternatives to meeting with a health visitor which was seen as expensive. Receiving this information from the midwife had the additional benefit of the two services being seen as working in partnership.

To be honest the information could probably be given quite easily in a letter in the post just setting out what a health visitor would do and what support they provide once you’ve had the baby. And I think that’s all it needed to be, just, essentially more information about what the health visitor does rather than actually your individual health visitor.

Katherine – no AC

Oh no, I think that’s absolutely fine for the midwife to have told me. Yeah, no, I don’t think I would have needed to speak to the health visitor. I think that would have been unnecessary.

Joanna – no AC

Not necessarily to see them but just, even for the midwife to explain there will be one and this is what happens in the first few weeks after having the baby. Because you have no idea what to expect, who you’re going to see.

Peta – no AC

Yeah. I do. I think that, maybe at one of the midwife appointments? Then they could give you a little leaflet or something because, or even just by post. But I imagine it would be easier and save a bit of money if your midwife would say, you’re about to be contacted, or do you want to contact them? Or whatever. I think knitting that together rather than them contacting you separately I think that, because also it sticks in your head. If you just get something through the door or a phone call it feels like, oh something else to kind of do, but yeah. I think that if they could somehow add that in to one of the midwife appointments, it would be good

Orla – no AC

Other participants, both those with or without experience of an antenatal health visitor contact, suggested a face-to-face meeting with their health visitor due to it being more personable and enabling rapport building.

No, I thought it was quite nice because if you’re just talking on the phone it’s a bit impersonal. She called me first before she came round, but it was nice to meet her because she was quite involved for the first few months of their lives, so it was good to see her first.

Anna – AC

I think meeting in person would be good. It’s just more personable, you know, and easier to get to know them if you’re talking to them in person rather than getting a letter in the post.

Leah – no AC

In terms of timing of the contact, participant reports were more consistent. Both women with and without antenatal contact agreed that contact in the third trimester was most appropriate. The most appropriate timing did, however, depend on whether the woman’s circumstances. Anna, for example, was expecting twins and had her visit as if she was expecting one child.

Sometime between, yeah about a month before he was born and him actually being born, I can’t remember exactly when… No I think that was the right time. I think any earlier and it might have been too early.

Beatrice – AC

I think she thought she was coming quite early and then she realised that I was having twins so she was a bit late, because, so I think if she was imagining I was going to be at 40 weeks, deliver at 40 weeks she was thinking, come at 35, 36 weeks, but actually I was delivering before 37, so she should have come a few weeks earlier.

Anna – AC

## Discussion

The overall finding from this study is a lack of clarity from mothers regarding the aim of the antenatal health visitor contact. This subsequently influences the information that women receive and want, and the mode and timing of contact. The aim of the antenatal contact is to assess the needs of the family before the birth and support preparation for parenthood, with additional care for those families who need it (Department of Health, 2009). However, participants’ experiences of this were mixed. These findings are discussed below; then, implications for practice are presented.

One of the aims of the antenatal contact is to build rapport with the woman and her family in preparation for parenthood and postnatal care. This relationship building was appreciated by several participants and this continuity of care from the antenatal to postnatal period is consistently wanted by women (Barimani *et al*., 2015). Two participants provided accounts of negative experiences. One participant felt she was being checked up on by the health visitor, and the other participant complained about the health visitor’s lack of knowledge. These negative experiences are consistent with other studies (Donetto *et al*., 2013). They suggest that women may not fully understand the aim of the contact, and that health visitors need to be aware of how their position of expertise may affect relationship building with mothers (Peckover and Aston, 2018).

The participants who had experience of an antenatal visit from a health visitor consistently told us their perception of the visit was to provide information about the health visiting service. Whilst this information was appreciated by many women, some participants questioned the value of receiving this information due to having had a previous baby or being told the same information postnatally. Whilst the first contact with a health visitor will involve some information giving (Bidmead *et al*., 2016) the main focus of the contact should be assessing the health and social needs of the family and supporting preparation for parenthood. Our findings suggest that this aim is not appropriately conveyed to women.

Participants who had not experienced antenatal health visitor contact mentioned confusion about the role of the health visitor and midwife and what to expect from the health visiting service. This is in line with English (Curtis and Davidson, 2013; Donetto *et al*., 2013) and international (Kurth *et al*., 2016) research where parents-to-be want information depicting the chain of care during and after pregnancy to help them understand what to expect when and from whom. This suggests that this is a world-wide problem other healthcare systems are yet to solve. Inaccurate knowledge and misconceptions about health visiting are likely to have important ramifications, such as women not making the most of the contact with the health visitor, and potentially feeling apprehensive about the first visit (Donetto *et al*., 2013). It is thus imperative that information concerning health visitors’ role and service remit is widely disseminated to ensure the antenatal contact can reach its full potential.

If women fully understood the aim of the antenatal contact it is unlikely they would suggest it could be conveyed in a letter, as some participants suggested in this study. The views on mode of contact varied between our participants, with other participants suggesting a midwife could provide the information about health visiting services. Whilst this would have the benefit of having midwifery and health visiting being seen as joined up which mothers want (Barimani and Hylander, 2012; Aquino *et al*., 2018), midwives should provide information on the health visiting service to parents without making the health visitor antenatal appointment superfluous. It is therefore concerning that there is still widespread confusion about the role of the health visitor (Aquino *et al*., 2016) which is hampering partnership working from happening in practice.

Home visits were experienced by most of our participants who had an antenatal contact and were also suggested by women who had not had this contact. Home visits are seen as personable and a good setting for relationship building (Tuominen *et al*., 2014). They are also convenient for women who prefer to meet a healthcare professional in their home or do not attend other services such as antenatal education. Home visiting is one of the ‘triad of core practices’ (the others being relationship building and needs assessment) identified in a review of research on health visiting that has been valued for more than 30 years (Cowley *et al*., 2013). Previous research confirms our findings as home visits are consistently well liked by parents-to-be (Kurth *et al*., 2016) although some women prefer meeting in a GP surgery or children’s centre (Curtis and Davidson, 2013).

Encouragingly, the participants reported wanting the health visitor antenatal contact in the third trimester, in line with current policy (Department of Health, 2009). For health visitors to know when to best contact women they need accurate and timely information from midwives. A recent evaluation of the antenatal contact found that the pregnancy notification from midwives to health visitors needs improvement (Monaghan and Owen, 2012) and our findings add to previous calls for better collaborative practice between midwives and health visitors (Aquino *et al*., 2016).

### Strengths and limitations

A few strengths and limitations regarding the current study must be noted. A considerable strength is the sample size as this is one of the largest studies on health visitor antenatal contact to date. Most areas of England were covered due to our social media recruitment which is crucial when exploring experiences of services that are likely to be fragmented. Interviewing both women with experience of antenatal contact and those without is a benefit as we were able to compare and contrast women’s views, thus strengthening our overall finding that women do not understand the aim of the antenatal contact.

Due to the current findings coming from a large interview study (exploring women’s views on collaboration between midwives and health visitors’ during and after pregnancy), few participants (*n* = 9) had experienced a face-to-face antenatal contact. More in-depth research is needed regarding the antenatal contact specifically with women who have had this contact and should ideally be done during pregnancy. A caveat must also be noted regarding women’s views on what they think they want, as it is always difficult to know what is helpful regarding care that has not been experienced. To avoid this as much as possible, we interviewed women postnatally to include the benefit of hindsight. Nevertheless, it is difficult to imagine what an antenatal contact may be like for women who never knew it was an option.

### Implications for practice

Our findings support previous policy and practice recommendations regarding the need for stronger links between midwifery care and health visiting (Aquino *et al*., 2016) to facilitate contact between health visitors and parents-to-be (Donetto *et al*., 2013). More specifically, it is clear that midwives are in a good position to inform women of the health visiting service, but they must first know themselves the remit of health visitors. Research suggests that this can be done through face-to-face training for midwives and health visitors (Olander *et al*., 2018a) or alternatively through midwifery pre-registration education (Brook et al., 2018; Olander *et al*., 2018b). That said, the information from midwives cannot be offered in lieu of an antenatal contact with health visitors. Currently, the understanding of the antenatal visit is poor, which impedes it reaching its potential in supporting pregnant women and their families. More research is needed to identify good practice regarding the antenatal contact, and to interview women experiencing and health visitors providing this contact. It is also important to research health visitors’ understanding of the antenatal contact, considering the calls for more training before this contact can be provided (Monaghan and Owen, 2012).

## Conclusion

The aim of the current study was to explore recent mothers’ experiences and views of antenatal health visitor contact. In addition to its relevance to current research and policy, this was highlighted as important by the members of our PPI group who had no contact with health visitors during pregnancy. The findings of this study are also internationally relevant: approximately one in five child and family health nurses (Australian equivalent of health visitor) has contact with women in the antenatal period (Psaila *et al*., 2014) and this practice is also reported in Sweden albeit anecdotally (Barimani and Hylander, 2012). Findings from this large interview study show that the health visitor role and subsequently the health visitor antenatal contact are poorly understood by women. This needs urgent attention, as it is not until better awareness of the health visiting service has been achieved that the antenatal contact can reach its true potential in supporting pregnant women and their families in preparing for parenthood.
